# Ecological Responses of Maize Rhizosphere to Antibiotics Entering the Agricultural System in an Area with High Arsenicals Geological Background

**DOI:** 10.3390/ijerph192013559

**Published:** 2022-10-19

**Authors:** Mengli Li, Yongshan Chen, Ying Feng, Xiaofeng Li, Lili Ye, Jinping Jiang

**Affiliations:** 1 College of Environmental Science and Engineering, Guilin University of Technology, Guilin 541006, China; 2 Guangxi Collaborative Innovation Center for Water Pollution Control and Water Safety in Karst Area, Guilin University of Technology, Guilin 541004, China; 3 School of Resources and Environmental Science, Quanzhou Normal University, Quanzhou 362000, China

**Keywords:** oxytetracycline, sulfadiazine, rhizosphere, enzyme activities, arsenite, arsenate

## Abstract

Metal(loid)s can promote the spread and enrichment of antibiotic resistance in the environmental ecosystem through a co-selection effect. Little is known about the ecological effects of entering antibiotics into the environment with long-term metal(loid)s’ resistance profiles. Here, cow manure containing oxytetracycline (OTC) or sulfadiazine (SA) at four concentrations (0 (as control), 1, 10, and 100 mg/kg) was loaded to a maize cropping system in an area with high a arsenicals geological background. Results showed that exogenous antibiotics entering significantly changed the nutrient conditions, such as the concentration of nitrate nitrogen, ammonium nitrogen, and available phosphorus in the maize rhizosphere soil, while total arsenic and metals did not display any differences in antibiotic treatments compared with control. Antibiotics exposure significantly influenced nitrate and nitrite reductase activities to reflect the inhibition of denitrification rates but did not affect the soil urease and acid phosphatase activities. OTC treatment also did not change soil dehydrogenase activities, while SA treatment posed promotion effects, showing a tendency to increase with exposure concentration. Both the tested antibiotics (OTC and SA) decreased the concentration of arsenite and arsenate in rhizosphere soil, but the inhibition effects of the former were higher than that of the latter. Moreover, antibiotic treatment impacted arsenite and arsenate levels in maize root tissue, with positive effects on arsenite and negative effects on arsenate. As a result, both OTC and SA treatments significantly increased bioconcentration factors and showed a tendency to first increase and then decrease with increasing concentration. In addition, the treatments decreased translocation capacity of arsenic from roots to shoots and showed a tendency to increase translocation factors with increasing concentration. Microbial communities with arsenic-resistance profiles may also be resistant to antibiotics entering.

## 1. Introduction

Antibiotic pollution of farmland soil has become a key factor influencing the sustainability of agricultural ecosystems [1,2]. The application of animal manure and manure-based fertilizers is the main way for antibiotics to enter the farmland soil, due to the high residual concentrations of antibiotics in animal feces or urine in intensive farming systems [3,4,5]. As a result, antibiotic molecules are increasingly found in the agricultural environment. One of the most noted consequences of antibiotic pollution is the increased frequency of antibiotic resistance/tolerance profiles, such as antibiotic resistance genes (ARGs) and antibiotic resistance-carrying bacteria (ARBs) [6,7,8], which may affect the natural microbial communities and lead to influences on the fundamental ecological processes or the maintenance of soil quality. In fact, antibiotic pollution has been involved in biogeochemical cycling and organic contaminant degradation in direct or indirect ways [9,10]. A consequent shift of their ecological functions in natural ecosystems is one of the most concerning topics in the scientific community. Rhizosphere is involved in biochemical processes such as the degradation of pollutants, the mineralization of organic matter, and nutrient elements cycle between soil and plant compartments, reflecting the quantitatively important components in terrestrial ecosystems.

There is evidence to suggest that metal contamination in the natural environment plays a role in the development and spread of antibiotic resistance. Metals such as arsenic, cadmium, chromium, copper, and lead have been linked to this phenomenon [11,12,13]. These co-selection mechanisms include different resistance determinants present on the same genetic element (co-resistance) and the same genetic determinant responsible for resistance to antibiotics and metals (cross-resistance) [14,15,16]. Co-resistance occurs when genes encoding for resistant phenotypes are situated on the same genetic structure, for example, a plasmid, transposon, or integrin [14]. Cross-resistance is another co-selection phenomenon that occurs when different antimicrobial agents attack the same target, initiate a common pathway to cell death, or share a common route of access to their respective targets [14]. Antibiotic resistance may be more likely to develop in environments where toxic metals accumulate to toxic levels. In fact, metal contamination has been linked to the increased abundance of antibiotic resistance genes in diverse environmental scenarios, especially in long-term field experiments [17]. Compared with man-made antibiotics in the soil environment, metals/metalloids existed earlier and may present above permissible concentrations to soil organisms, due to significant anthropogenic emission. That is, the presence of soil metals/metalloids can exert long-term selection pressure on the soil ecosystem, resulting in the formation of resistance/tolerance profiles or adaptability [18,19].

Arsenic is one of the most common elements in the Earth’s crust, and widely distributed in the environment matrices, which poses negative impacts on terrestrial ecosystems [20]. Relatively high concentrations of naturally-occurring arsenic can occur in high geological background soil environments and provides selective pressure on microbes to maintain resistance/tolerance profiles [21,22,23]. This adaptability in environmental reservoirs with high arsenic concentrations may contribute to selecting antibiotic-resistant strains or increasing the proliferation of antibiotic resistance via several co-selection mechanisms [24,25]. Significant antibiotic residuals in animal manure or manure-based fertilizers have been considered as implications of antibiotics in terms of the risks to soil ecosystems [26,27]. However, the ecological responses of high geological background areas with arsenicals to antibiotics entering remains to be investigated.

In this work, pot experiments were set to assess the ecological responses to antibiotics entering in an agricultural system in an area with a high arsenicals geological background. The ecological effects of soil enzyme activities, rhizosphere processes, and arsenic transformation were combined to define the ecological impacts.

## 2. Materials and Methods

### 2.1. Soils and Organic Fertilizer Preparation

Soil samples were collected from an area with a high arsenicals geological background in Guangxi Zhuang Autonomous Region, south China. Total As concentration of soil samples was 124.57 mg/kg, which exceeded the national soil pollution risk control standard of China (GB 15618-2018) by 211.43%. Other metal concentrations such Cu (39.62 mg/kg), Pb (40.96 mg/kg), Zn (112.50 mg/kg), Ni (34.39 mg/kg), and Cr (72.26 mg/kg) were lower than the standard range. The physiochemical properties of soil samples were loam soil with a pH value of 5.1 and contained 12.51 g/kg organic matter, 83.40 mg/kg total nitrogen, 30.19 mg/kg nitrate nitrogen, 17.63 mg/kg ammonium nitrogen, 98.81 mg/kg total organic carbon, and 9.95 mg/kg available phosphorus. The manure-based fertilizer used in the experiment was cow dung, which was from the agro-pastoral area of Inner Mongolia Autonomous Region, North China. The pH of the organic fertilizer was 6.2, the organic matter was 30.98 g/kg, the As concentration was 2.75 mg/kg, and no antibiotics were detected in the fertilizer. The manure-based fertilizer was dried, then passed through a 2.0 mm sieve, and finally treated with oxytetracycline (OTC) or sulfadiazine (SD) at four concentrations of 0 (as control), 1, 10, and 100 mg/kg, to simulate organic fertilizer contaminated with different antibiotics and exposure concentrations. The tested corn variety was Jingkenuo 2000, provided by Beijing Huaao Nongkeyu Breeding Development Co., Ltd. (Beijing, China).

### 2.2. Pot Experiments Design

Soil collected from a high geological background area with arsenicals was also dried, and then passed through a 2.0 mm sieve. Soil (1500 g) was mixed homogeneously with 150 g treated manure-based fertilizer (treated with OTC or SD at four concentrations, respectively). The soil without manure-based fertilizer served as the blank control (CK), and with manure-based fertilizer at 0 mg/kg antibiotic concentration served as treatment control (CK0). There are eight treatments with five replicates each as follows: blank control (CK), soil with manure-based fertilizer at 0 mg/kg antibiotic concentration (CK0), soil with manure-based fertilizer at 1 mg/kg OTC concentration (OTC1), soil with manure-based fertilizer at 10 mg/kg OTC concentration (OTC10), soil with manure-based fertilizer at 100 mg/kg OTC concentration (OTC100), soil with manure-based fertilizer at 1 mg/kg SD concentration (SD1), soil with manure-based fertilizer at 10 mg/kg SD concentration (SD10), and lastly, soil with manure-based fertilizer at 100 mg/kg SD concentration (SD100). These eight treatment soils were then loaded into plastic pots (23.0 cm × 31.5 cm, inner diameter × height) and kept at soil water capacity of 60% for 7 days balance. The corn seeds were disinfected with 10% hydrogen peroxide for 30 min, rinsed with distilled water, and incubated at 25 °C until germination. Corn seedlings that were approximately 15.0 cm in height and have 3 leaves were transplanted to the treatment soils. They were then grown in a controlled greenhouse that is located in Guilin city. The temperature in the greenhouse was kept at 20–30 °C and the relative humidity of the air was kept at 70–85%. Light intensity and time was not controlled in the greenhouse. The pots were irrigated early in the morning to maintain enough water for plant growth. After 100 days of growth, the plants were harvested to separate the roots and rhizosphere soil for the next step analysis.

### 2.3. Soil Enzyme Activities

Soil urease activity was determined according to the sodium phenate–sodium hypochlorite colorimetry method and expressed as the number of milligrams of NH_4_^+^-N generated in each gram of soil after 24 h of incubation [28]. Dehydrogenase activity (DHA) was assessed by the triphenyltetrazolium chloride (TTC) colorimetric method and expressed as the production of the triphenylformine generated in each gram of soil as an enzyme activity unit [29]. Acid phosphatase activity was measured by the disodiumphenyl phosphate colorimetric method and expressed as phenol per gram of dry soil in 24 h at 37 °C [29].

Soil nitrate reductase and nitrite reductase activities were determined using the Soil Nitrate Reductase (NR) Activity Assay Kit and the Soil Nitrite Reductase Activity Assay Kit (Solarbio Science & Technology Co., Ltd., Beijing, China). Soil nitrate reductase catalyzes the reduction of nitrate to nitrite, and the generated nitrite can quantitatively generate red azo compounds with p-aminobenzenesulfonic acid and α-naphthylamine under acidic conditions. The unreacted NADH will inhibit the subsequent color reaction, and then carry out the subsequent reaction with PMS; the generated red azo compounds are 520 nm and have a maximum absorption peak, which can be determined by spectrophotometry. Nitrite reductase can reduce NO_2_^−^ to NO and reduce the NO_2_^−^ in the sample to participate in the diazotization reaction to produce a purple-red compound, that is, the change in absorbance at 540 nm can reflect the activity of nitrite reductase in soil. The detail procedures of these two assay kits are provided in the supporting information. One unit of nitrate reductase activity is defined as the amount of enzyme that catalyzes the production of 1 μmol of NO_2_^−^ per every gram of soil per day; while one unit of nitrite reductase activity is defined as the amount of enzyme catalyzes the reduction of 1μmol NO_2_^−^ per every gram of soil per day.

### 2.4. Soil Physiochemical Properties

The soil pH was measured in a soil/deionized water slurry at a ratio of 1:2.5 using a pH-EC meter (Accumet Excel XL60, Fisher Scientific Inc., Hampton, NH, USA). Soil available phosphorus was extracted by hydrochloric acid ammonium fluoride and determined by molybdenum antimony colorimetry. Soil nitrate nitrogen (NO_3_^−^-N) and ammonium nitrogen (NH_4_^+^-N) were extracted by 0.01 mol/L anhydrous calcium chloride and quantified using a Flow Injection Autoanalyzer simultaneously. Soil total carbon content (TC), total nitrogen content (TN), and total sulfur content (TS) were measured using an Elementar Analysensysteme GmbH (Vario MAX, Langenselbold, Germany).

Soil metals such as Pb, Cr, Zn, Ni, Cu, and As were digested by microwave-assisted acid digestion using trace-pure HNO_3_ (2.5 mL) and HF (1.5 mL) and a closed-vessel high-pressure microwave digester—Multiwave GO (Anton Paar, Graz, Austria) [30], and finally determined by ICP-OES (Optima 7000 DV, PerkinElmer, Waltham, MA, USA), except As. Total As concentration was determined by an atomic fluorescence spectrometer (SA-20, Beijing Titan Instrumentals Co. Ltd., Beijing, China).

Extraction of individual species of As (As^III^ and As^V^) was based on GB/T 5009.11-2014. In brief: 1.0 g of homogenized soil or root sample was extracted with 20 mL of 0.15 mol/L nitric acid for overnight, and then thermally extracted in a 90-degree incubator for 2.5 h with shaking for 1 min every 0.5 h. Extraction solution was centrifuged at 8000× *g* for 15 min and filtered through a 0.45 μm cellulose acetate disk filter for an atomic fluorescence spectrometry speciation analyzer. Arsenic separation and instrument preformation was described in one of our previous works [30].

### 2.5. Data Analysis

Analysis of variance (ANOVA) was employed to find out if there is a difference in ecological indicators such as enzyme activities, element concentration, and arsenic species between treatments and control. Tukey’s Highly Significant Differences (HSD) was applied as a post hoc test for means. Factorial ANOVA was conducted for different monitoring indicators at exposure concentration and antibiotic type, respectively, of each sediment type with Tukey’s HSD test for means. All statistical analyses were conducted by the statistical software package SPSS version 23.0 for Windows (SPSS Inc., Chicago, IL, USA). Redundancy analysis (RDA) was used to explore possible relationships between the responses of enzyme activities and basic properties (e.g., pH, EC, and TC) in all treatments and was conducted by Canoco 5.0 software (Microcomputer Power, Ithaca, NY, USA).

## 3. Results

### 3.1. Ecological Effects of Antibiotics on Rhizosphere Processes

**Figure 1:**
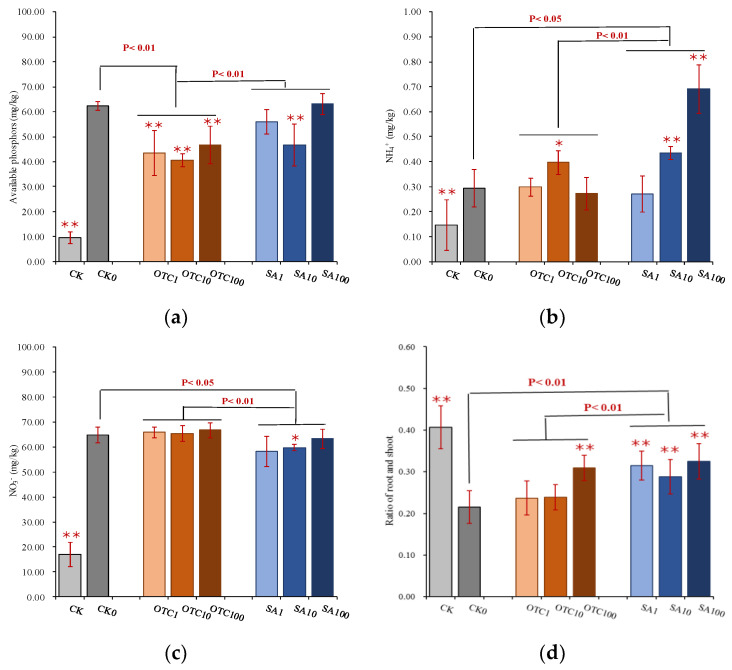
Changes of soil nutrient factors of rhizosphere soil in different treatments with or without plants. (**a**) Soil available phosphorus concentration; (**b**) Soil NH_4_⁺-N concentration; (**c**) Soil NO_3_^−^-N concentration; (**d**) Ratio of root and shoot. CK represents the soil without manure-based fertilizer; CK0 is the soil with manure-based fertilizer at 0 mg/kg antibiotic concentration; OTC1 is the soil with manure-based fertilizer at 1 mg/kg OTC concentration; OTC10 shows the soil with manure-based fertilizer at 10 mg/kg OTC concentration; OTC100 is the soil with manure-based fertilizer at 100 mg/kg OTC concentration; SA1 is the soil with manure-based fertilizer at 1 mg/kg SA concentration; SA10 indicates the soil with manure-based fertilizer at 10 mg/kg SA concentration; SA100 is the soil with manure-based fertilizer at 100 mg/kg SA concentration. * or ** represents significant differences (*p*  <  0.05 or *p* < 0.01) between each antibiotic treatment (e.g., OTC1, OTC10) and CK0, and *p*  <  0.05 or *p* < 0.01 on the line represents significant differences between treatment groups such as control, OTC, and SA treatments. NH_4_^+^ stands for soil ammonium nitrogen concentration; NO_3_^−^ represents the soil nitrate nitrogen concentration.

**Figure 2:**
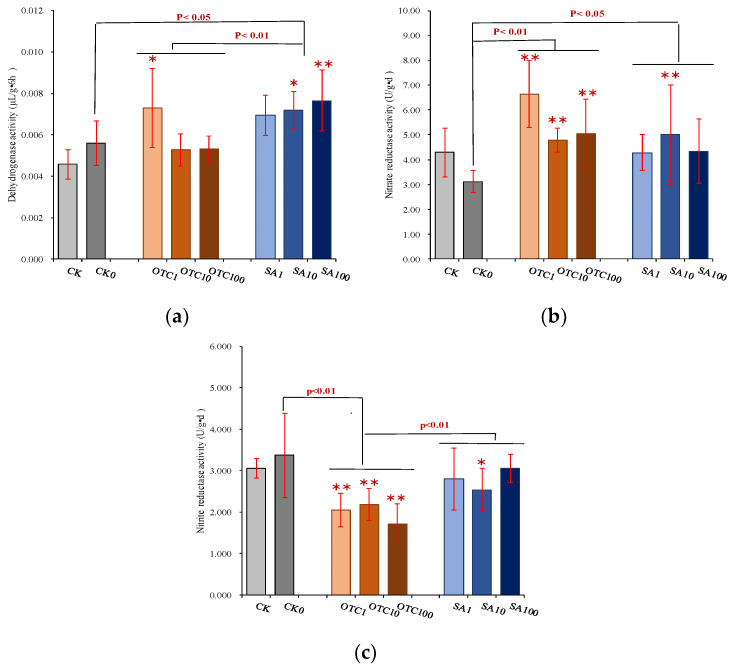
Changes of soil enzyme activities of rhizosphere soil with different treatments. (**a**) Soil dehydrogenase activity; (**b**) Soil nitrate reductase activity; (**c**) Soil nitrite reductase activity. CK is the soil without manure-based fertilizer; CK0 shows the soil with manure-based fertilizer at 0 mg/kg antibiotic concentration; OTC1 is the soil with manure-based fertilizer at 1 mg/kg OTC concentration; OTC10 is the soil with manure-based fertilizer at 10 mg/kg OTC concentration; OTC100 indicates the soil with manure-based fertilizer at 100 mg/kg OTC concentration; SA1 is the soil with manure-based fertilizer at 1 mg/kg SA concentration; SA10 is the soil with manure-based fertilizer at 10 mg/kg SA concentration; SA100 presents the soil with manure-based fertilizer at 100 mg/kg SA concentration. * or ** represents significant differences (*p*  <  0.05 or *p*< 0.01) between each antibiotic treatment (e.g., OTC1, OTC10) and CK0; and *p*  <  0.05 or *p*< 0.01 on the line represents significant differences between treatment groups such as control, OTC, and SA treatments.

The application of manure fertilizer significantly improved the soil environmental quality, as evidenced by the increased concentrations of nutrients such as ammonium nitrogen, nitrate nitrogen, and available phosphors. Plants also contributed significant effects on soil nutrient conditions because there was a significant decreasing of these parameters in rhizosphere soil compared with the soil treatment without plant growth (Figure S1 in Supplementary). In rhizosphere soil (Figure 1), OTC treatment significantly decreased the concentration of soil available phosphors and did not display any difference with increased exposure concentration. SA treatment resulted in a significant decrease in the concentration of soil available phosphors at the 10 mg/kg concentration. OTC treatment did not reflect significant variances of the ammonium nitrogen concentration in rhizosphere soil, compared with the CK0, except for that at the OTC concentration of 10 mg/kg. However, SA treatment caused a significant increase in the concentration of ammonium nitrogen. For nitrate nitrogen, SA treatment showed a significant decrease in the concentration of nitrate nitrogen in rhizosphere soil, in particular at 10 mg/kg concentration of SA treatment, while OTC did not change the concentration of nitrate nitrogen. For other soil chemical properties such as pH, total arsenic, and heavy metals in the rhizosphere, both OTC and SA treatment did not present any variances compared with the control (CK0, Figure S2 in Supplementary). But for exogenous substances, SA treatment greatly influenced the root growth, shown as a significant increase in the root-shoot ratio of maize, although with no differences when exposure concentration increased (Figure 2). Root-shoot ratio also significantly increased at the 100 mg/kg concentration of OTC treatment.

### 3.2. Ecological Effects of Antibiotics on Soil Enzyme Activities

Dehydrogenase is an important enzyme that reflects the activity of microorganisms and the state of organic matter in the sediment ecosystem. In this work, dehydrogenase activities did not change significantly with the presence of OTC exposure, except for an increase with 1 mg/kg OTC concentration in manure fertilizer (Figure 2). Compared with CK0, dehydrogenase activities showed a significant increase in SA treatment, and indicated a tendency to increase with exposure concentration. The variance of dehydrogenase activities promoted by SA at the three exposure concentrations was higher than that of the OTC. Urease activities and acid phosphatase activities did not display any difference when entering antibiotics, except for significantly decreasing urease activities at the SA concentration of 100 mg/kg (Figure S3 in Supplementary). However, nitrate reductase and nitrite reductase activities demonstrated significant variances with exposure to antibiotics (Figure 1). The nitrate reductase, which catalyzes the first reaction in nitrate assimilation, was significantly promoted by antibiotic presence, in particular at OTC exposure treatment. A low exposure concentration (1 mg/kg in manure fertilizer) of OTC had the highest increment effects in all treatments compared with CK0 (0 mg/kg antibiotics in manure fertilizer) and showed a tendency to decrease with increasing concentration. SA treatment also significantly increased nitrate reductase activities and showed a tendency to first increase and then decrease with increasing concentration. The nitrite reductase, a key enzyme in the dissimilatory denitrification chain, was significantly inhibited by OTC exposure, but did not display any difference with increased exposure concentration. Nitrite reductase activities did not change significantly with an increasing SA antibiotic concentration compared with CK0, with the exception of a decrease for SA concentration at 10 mg/kg in manure fertilizer. The inhibition effects of nitrite reductase activities by OTC exposure were significantly higher than those of SA exposure.

### 3.3. Ecological Effects of Antibiotics on Arsenic Mobilization

**Figure 3:**
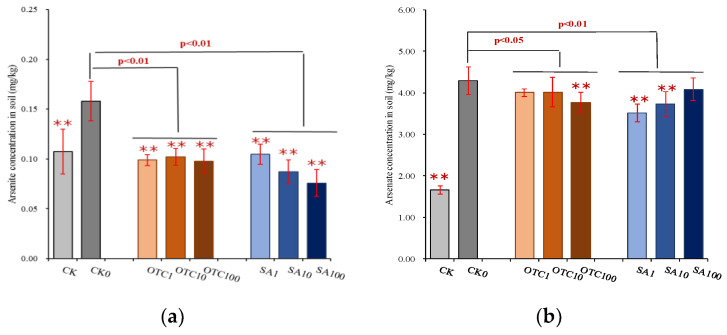

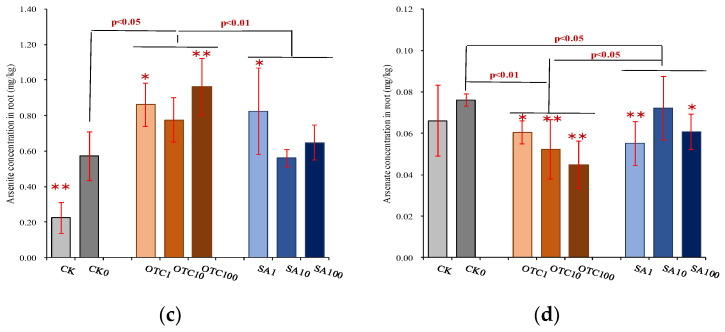
Changes in arsenic mobilization of rhizosphere soil in different treatments. (**a**) Arsenite concentration in soil; (**b**) Arsenate concentration in soil; (**c**) Arsenite concentration in root; (**d**) Arsenate concentration in root. CK is the soil without manure-based fertilizer; CK0 shows the soil with manure-based fertilizer at 0 mg/kg antibiotic concentration; OTC1 is the soil with manure-based fertilizer at 1 mg/kg OTC concentration; OTC10 is the soil with manure-based fertilizer at 10 mg/kg OTC concentration; OTC100 is the soil with manure-based fertilizer at 100 mg/kg OTC concentration; SA1 is the soil with manure-based fertilizer at 1 mg/kg SA concentration; SA10 is the soil with manure-based fertilizer at 10 mg/kg SA concentration; SA100 is the soil with manure-based fertilizer at 100 mg/kg SA concentration. * or ** represents significant differences (*p*  <  0.05 or *p* < 0.01) between each antibiotic treatment (e.g., OTC1, OTC10) and CK0; and *p*  <  0.05 or *p* < 0.01 on the line represents significant differences between treatment groups such as control, OTC, and SA treatments.

**Figure 4:**
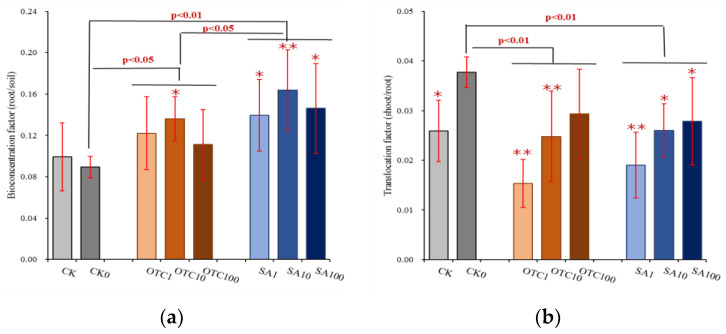
Changes in arsenic translocation in maize cropping system with antibiotics treatments. (**a**) Bioconcentration factor; (**b**) Translocation factor. CK is the soil without manure-based fertilizer; CK0 is the soil with manure-based fertilizer at 0 mg/kg antibiotic concentration; OTC1 is the soil with manure-based fertilizer at 1 mg/kg OTC concentration; OTC10 is the soil with manure-based fertilizer at 10 mg/kg OTC concentration; OTC100 is the soil with manure-based fertilizer at 100 mg/kg OTC concentration; SA1 is the soil with manure-based fertilizer at 1 mg/kg SA concentration; SA10 is the soil with manure-based fertilizer at 10 mg/kg SA concentration; SA100 is the soil with manure-based fertilizer at 100 mg/kg SA concentration. * or ** represents significant differences (*p*  <  0.05 or *p* < 0.01) between each antibiotic treatment (e.g., OTC1, OTC10) and CK0; and *p*  <  0.05 or *p* < 0.01 on the line represents significant differences between treatment groups such as control, OTC, and SA treatments.

The total concentration of arsenic in rhizosphere soil did not change with the antibiotic treatments. However, extracted arsenite concentrations in rhizosphere soil were significantly decreased by both OTC and SA treatment, and showed a tendency to decrease with increasing concentration of SA (Figure 3). Extracted arsenate concentrations were also significantly decreased by both OTC and SA treatment and showed a tendency to increase with an increasing concentration of SA. The variances of decreased effects of extracted arsenite concentrations by both OTC and SA treatments were significantly higher than extracted arsenate concentrations decreased by the two antibiotics. Extracted arsenite concentrations in maize roots were significantly increased by OTC treatment and did not display any differences with exposure concentration. SA treatment did not significantly change the concentration of extracted arsenite in roots compared with the CK0, except for at the SA concentration of 1 mg/kg. Both OTC and SA treatment significantly decreased the extracted arsenate concentrations in maize roots and showed a tendency to decrease with increasing concentration of OTC, and a tendency to first increase and then decrease with raising concentration of SA. The variances in the decreased effects of arsenate concentrations by OTC treatment were higher than those by SA treatment. Antibiotics significantly increased the arsenic uptake in high geological background area with arsenicals, as higher bioconcentration factors (ratio of root and soil) were obtained at antibiotics treatment compared with control (Figure 4). Both OTC and SA treatment significantly increased bioconcentration factors and showed a tendency to first increase and then decrease with increasing concentration. These promotion effects in SA treatment were higher than that in OTC treatment. On the contrary, both OTC and SA treatment significantly decreased translocation capacity of arsenic from roots to shoots and showed a tendency to increase translocation factors with increasing concentration (Figure 4). There were not any differences in the inhibition effects between OTC and SA treatments.

## 4. Discussion

Antibiotics existence can change the microbial community, as well as biomass, and inhibit the growth of target microorganisms. Microbial activity performs biogeochemical processes in ecosystems to services such as nutrient cycling, organic matter production, and turnover or degradation of pollutants regulated by microbial metabolism [31]. Thus, exogenous antibiotics exposure may pose a change to community structure and ecosystem functioning that could lead to side-effects on a biogeochemical process, in particular in the rhizosphere, which is a complex environment where roots interact with physical, chemical, and biological properties of soil [32]. In this work, nutrient parameters such as the concentration of nitrate nitrogen, ammonium nitrogen, and available phosphorus in rhizosphere soil display significant differences with antibiotic exposure. The impact of microbial activity and its community structure under antibiotics pressure may be one of the reasons, in particular with the biogeochemical cycle of elements such as biogeochemical N cycling [31,33]. The higher concentrations of ammonium nitrogen and lower concentrations of nitrate nitrogen in rhizosphere soil with antibiotic treatments were consistent with the inhibition effects of denitrification rates (defined as nitrate and nitrite reductase activities in this study). This was similar to the results that nitrogen cycling, such as nitrification and anammox, appeared to be less sensitive to antibiotic exposure (sensitivity at therapeutic concentrations) than denitrification [34,35]. Plant roots can interact with soil microorganisms to influence nutrient availability and uptake, which can in turn promote plant growth [32]. This explained the variances in nutrient conditions and roots/shoots ratios in antibiotics treatment compared with free antibiotics control (CK0, Figure 2). However, both OTC and SA treatments did not display any variances in the presence of most monitored substances, such as pH, total arsenic, and heavy metals in rhizosphere soil (Figure S3, Supplementary). The low responsiveness to antibiotics may be caused by the sustaining of resistance/tolerance profiles from a high geological background. Another possible reason is that these substances do not play a dominant role in the interaction between plant roots and microorganisms.

Soil enzymes are a sensitive biological/biochemical indicator of microbial activity to evaluate the ecological responses of microorganisms to environmental stress [36,37,38]. Enzyme activity can be influenced by antibiotics contamination in many environmental matrices, but the reaction behavior was controlled by exposure concentration, antibiotic type, exposure time, and environmental properties [39]. In correspondence with previous findings that the ecological response of enzyme activity to antibiotics is controlled by exposure concentration and antibiotic type, our results also demonstrated that the five targeted enzyme activities showed differences in response to entering antibiotics into a high geological background soil environment. Dehydrogenase activities did not change significantly with OTC treatment but were significantly promoted by SA treatment and showed a tendency to increase with exposure concentration. Similar effects of dehydrogenase activities to OTC were also observed in previous results, even at a concentration of 1000 mg/kg [39,40]; and stimulated response to SA after repeated antibiotic exposure [39,41]. Urease activity was not significantly affected by the two antibiotics entered. This is in contrast to previous findings that urease activity may be promoted initially upon exposure to antibiotics, but then inhibited with prolonged exposure [42,43]. Soil phosphatases, which play a major role in the mineralization processes of organic phosphorus substrates, also did not change with antibiotics treatments in the soil from a high geological background area with arsenics. This was dissimilar to the previous results that soil phosphatase activity was inhibited at the concentration range between 1 to 300 mg/kg during 22 days’ incubation [44]. The formation of resistance/tolerance profiles of microbial communities to high concentrations of arsenics in areas with a high geological background may be one of the reasons behind the low responsiveness of these two enzyme activities to antibiotics exposure, because of the frequent occurrence of co-resistance or cross-resistance of co-selection/tolerance profiles between metals and antibiotics [45,46]. These co-selection profiles may enhance in the situation where both metals and antibiotic resistance genes located on the same genetic element or the same resistance mechanism give resistance to both metals and antibiotics [16,45,46]. Through co-selection, microorganisms have recently demonstrated the ability to adapt to such hostile environments. For example, increased antibiotic resistance gene abundances were found to be highly correlated with trace metals (such as chromium, copper, nickel, lead, and iron) present in the soil [14,46]. Since the existence of metals resistance has been more pronounced in the bacterial genome over a longer time, the influence of existing metal contamination may be selecting for overall more resistant/fit bacterial communities. However, two nitrogen metabolism enzymes, nitrate reductase and nitrite reductase, seemed more sensitive to antibiotics treatment, shown as positive effects of antibiotics exposure on nitrate reductase activities and negative effects of antibiotics exposure on nitrite reductase activities. This was consistent with previous reports that antibiotics exposure inhibited denitrification rates, because nitrite reductase is a rate-limiting enzyme in the denitrification process [47,48,49,50]. Additionally, soil properties, such as carbon and nitrogen levels, may play a role in the microbial activity responsible for decomposition and other enzyme-related processes.

Soil microbes can influence arsenic mobilization and transformation by reduction (arsenate to arsenite) and oxidation (arsenite to arsenate) reactions to strengthen arsenite mobility or arsenate adsorption in the soil environment [51,52]. Both of the two tested antibiotics decreased the concentration of arsenite and arsenate in rhizosphere soil, but the inhibition effects of arsenite were higher than those of arsenate. This was similar to the results that aerobic arsenate reduction was not affected by antibiotics, but aerobic arsenite oxidizing bacteria were sensitive to antibiotic (i.e., chloramphenicol) exposure [53]. It is known that metals have a more significant impact on antibiotic resistance gene (ARG) profiles than detected antibiotics by inducing metal resistance, as well as selection processes of ARGs [54,55], suggesting that most arsenic-resistant bacteria may also be resistant to antibiotics. In this work, the tested soil was from a high geological background area with arsenicals, in which soil microbes had formed arsenic-resistant/tolerance profiles during geological selection. The changed concentrations of soil arsenite and arsenate in rhizosphere with antibiotic treatments may be due to the uptake by plants, because bioconcentration factors (root/soil) in antibiotic treatments were higher than those in free antibiotics control (CK0). The concentration of arsenite and arsenate in maize root tissue was also affected by treatment with antibiotics, with positive effects on arsenite and negative effects on arsenate. Plant roots are capable of rapidly taking up arsenite from the external medium, and arsenate is rapidly reduced to arsenite in root cells [56,57]. This is why the concentration of arsenite in root tissue is much higher than that of arsenate.

**Figure 5:**
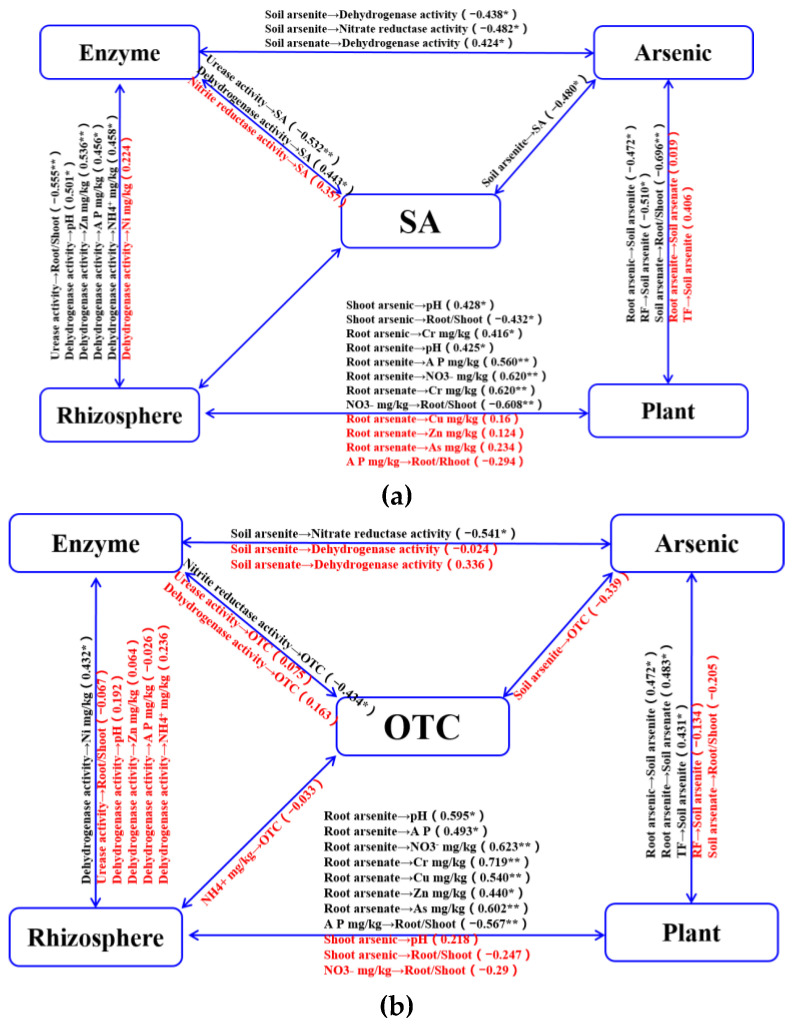
Relationships among biogeochemical processes of ecosystems and antibiotics treatments. (**a**) Relationships among biogeochemical processes of ecosystems and SA treatments; (**b**) Relationships among biogeochemical processes of ecosystems and OTC treatments. OTC is the oxytetracycline treatment; SA is the sulfadiazine treatment; A P is the concentration of soil available phosphorus; NH_4_^+^ stands for soil ammonium nitrogen concentration; NO_3_^−^ represents the soil nitrate nitrogen concentration; TF (translocation factor) is a ratio of arsenic concentrations in plant shoots to arsenic concentrations in plant roots; BF (bioconcentration factor) is a ratio of arsenic concentrations in plant roots to arsenic concentrations in soil; * or ** represents significant correlation (*p*  <  0.05 or *p* < 0.01). The black letters represent significant differences between biogeochemical processes and rhizosphere antibiotic treatment relationships in ecosystems, while the red letters represent no significant differences.

As antibiotic reservoirs, soil environments where biological effects occur are a complex array of soil properties mixed with a diverse array of residual antibiotics. Several studies have found that ecological responses are significantly linked to the physical and chemical characteristics of the environment, such as organic matter and soil texture [58], which may control the initial biological activity of the soil [8,59]. The adsorption of antibiotics on the mineral phase of soil has important consequences, not only for their mobility and stabilization but also for their bioavailability and bioaccessibility during interactions between antibiotics and microorganisms [43]. Further, soil with suitable texture and high organic carbon or nitrogen content may present higher microbial activity to resist environmental stress [60,61]. From our results, bacteria that are resistant to arsenic that forms in a high geological background may also be resistant to antibiotics, because of weak relationships with biogeochemical processes of ecosystems and antibiotics treatments in the rhizosphere (Figure 5). This could be explained by the facts that soil microbial community were primarily determined by high arsenic background; and the ability of microbes in arsenic-polluted soil to co-resist, cross-resist, co-regulate, or biofilm-induce the antibiotics presence [62,63]. Meanwhile, organic arsenic may be a primordial antibiotic, and the host in arsenic-rich conditions can pose evolutionary dynamics of host–microbe–environment interactions and present existence of a novel detoxification and adaptation mechanism [64,65]. However, the timing interaction of antibiotics with the soil environment could reduce this promotion effect and alter the microbial processes [66,67]. Microbial parameters, such as nutrient metabolism, enzymatic activities, and arsenic mobilization could be influenced by various factors, and they may not be specific for antibiotic effects in soil.

## 5. Conclusions

This study clarified the ecological response of maize rhizosphere soil to antibiotics entering the agricultural system in an area with a high arsenicals geological background. The results showed that (1) nutrient conditions, such as the concentration of nitrate nitrogen, ammonium nitrogen, and available phosphorus in rhizosphere soil, were significantly influenced by antibiotic treatment, while pH, total arsenic, and heavy metals demonstrated low responsiveness to antibiotics exposure. (2) Two enzymes involved in nitrogen metabolism, nitrate reductase and nitrite reductase, seemed more sensitive to antibiotics treatment, with positive effects on nitrate reductase activities and negative effects on nitrite reductase activities, while soil urease and phosphatases appeared to be resistant or tolerant to antibiotics treatment. (3) The concentration of arsenite and arsenate in the rhizosphere soil and in the maize root tissue varied with the antibiotic treatments, depending on the type of antibiotic and exposure concentration, resulting in the promotion of bioconcentration factors from soil to roots and inhibition of translocation factors from roots to shoots in maize growth. Arsenic-resistant bacteria formed in a high geological background possibly contributed to the low responsiveness of ecological effects to antibiotics entering, but various factors of the soil environment could also influence the microbial activities.

## Data Availability

Not applicable.

## Supplementary Materials

The following supporting information can be downloaded at: https://www.mdpi.com/article/10.3390/ijerph192013559/s1, Figure S1: Changes of soil nutrient factors of rhizosphere soil and bulk soil in different treatments with or without plants; Figure S2: Changes of soil pH, total arsenics and metals of rhizosphere soil and bulk soil in different treatments with or without plants; Figure S3: Changes of soil enzyme activities of rhizosphere soil with different treatments.
