# Supplementary material for: Ecological Responses of Maize Rhizosphere to Antibiotics Entering the Agricultural System in an Area with High Arsenicals Geological Background
Source: Int J Environ Res Public Health. 2022 Oct 19;19(20):13559. doi: 10.3390/ijerph192013559 (PMC9603512; doi:10.3390/ijerph192013559)
Supplement: Supplementary file 1 [file ijerph-19-13559-s001.zip › ijerph-1918104 supplementary for XML.pdf]

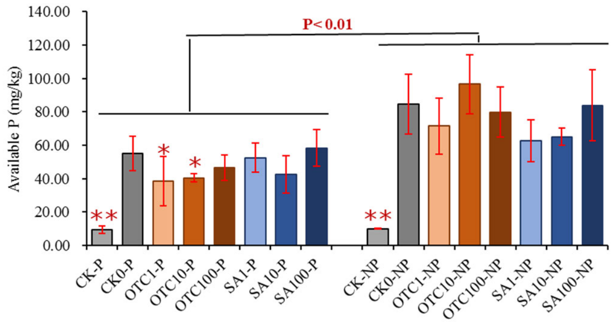

(a)

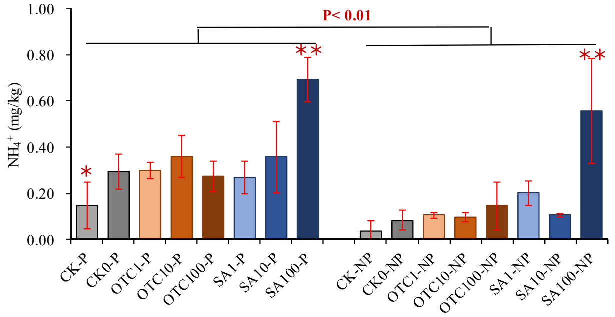

(b)

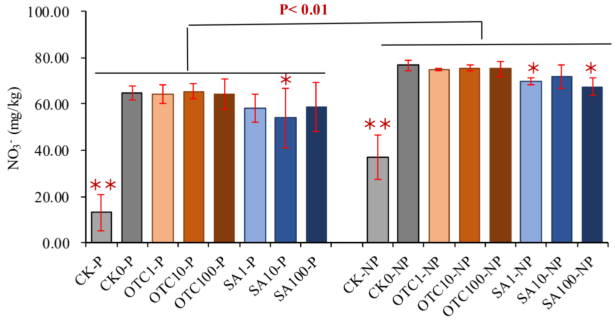

(c)

Figure S1. Changes of soil nutrient factors of rhizosphere soil and bulk soil in different treatments with or without plants.(**a**) Soil available phosphorus concentration ; (**b**) Soil NH_4^+-N concentration ; (**c**) Soil NO_3^--N concentration . CK represents the soil without manure-based fertilizer; CK0 is the soil with manure-based fertilizer at 0 mg/kg antibiotic concentration; OTC1 is the soil with manure-based fertilizer at 1 mg/kg OTC concentration; OTC10 shows the soil with manure-based fertilizer at 10 mg/kg OTC concentration; OTC100 is the soil with manure-based fertilizer at 100 mg/kg OTC concentration; SA1 is the soil with manure-based fertilizer at 1 mg/kg SA concentration; SA10 indicates the soil with manure-based fertilizer at 10 mg/kg SA concentration; SA100 is the soil with manure-based fertilizer at 100 mg/kg SA concentration. Suffix "-P" means the treatments with plants, and suffix"-NP" means the treatments without plants. * or ** represents significant differences (p < 0.05 or p< 0.01) between each antibiotic treatments (e.g., OTC1, OTC10) and CK0, and p < 0.05 or p< 0.01 on the line represents significant differences between treatment groups such as control, OTC, and SA treatments.

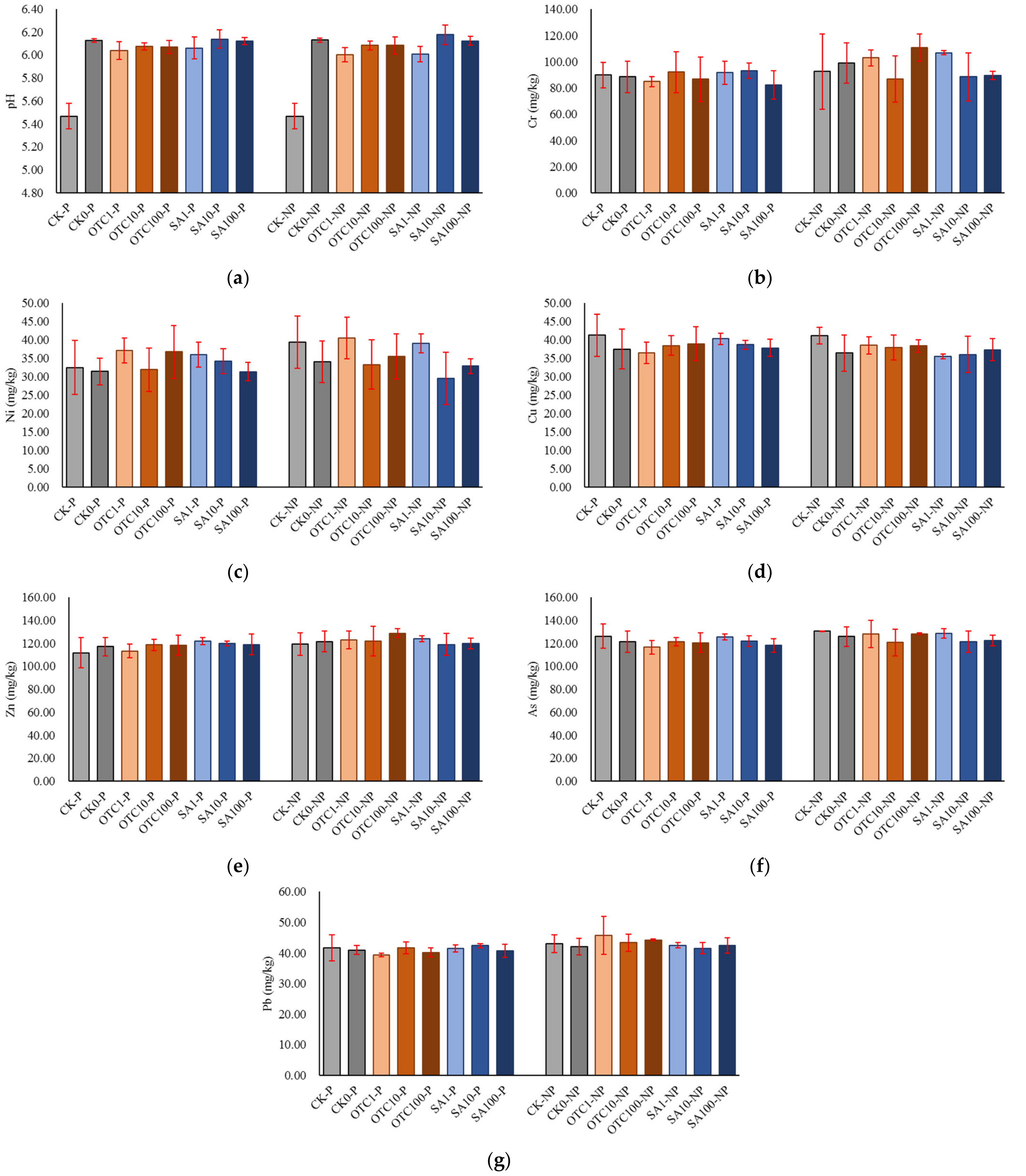

Figure S2. Changes of soil pH, total arsenics and metals of rhizosphere soil and bulk soil in different treatments with or without plants. (**a**) Soil pH Value; (**b**) Chromium concentration in soil; (**c**) Nickel concentration in soil; (**d**) Cuprum concentration in soil; (**e**) Zinc concentration in soil; (**f**) Arsenic concentration in soil; (**g**) Lend concentration in soil. CK represents the soil without manure-based fertilizer; CK0 is the soil with manure-based fertilizer at 0 mg/kg antibiotic concentration; OTC1 is the soil with manure-based fertilizer at 1 mg/kg OTC concentration; OTC10 shows the soil with manure-based fertilizer at 10 mg/kg OTC concentration; OTC100 is the soil with manure-based fertilizer at 100 mg/kg OTC concentration; SA1 is the soil with manure-based fertilizer at 1 mg/kg SA concentration; SA10 indicates the soil with manure-based fertilizer at 10 mg/kg SA concentration;

SA100 is the soil with manure-based fertilizer at 100 mg/kg SA concentration. Suffix "-P" means the treatments with plants, and suffix"-NP" means the treatments without plants. * or **represents significant differences ($p < 0.05$ or $p < 0.01$) between each antibiotic treatments (e.g., OTC1, OTC10) and CK0, and $p < 0.05$ or $p < 0.01$ on the line represents significant differences between treatment groups such as control, OTC, and SA treatments.

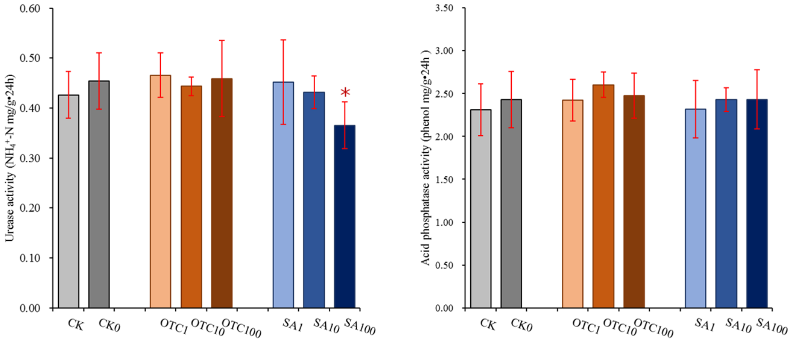

(**a**) (**b**)

Figure S3. Changes of soil enzyme activities of rhizosphere soil with different treatments. (**a**) Soil urease activity; (**b**) Soil acid phosphatase activity. CK represents the soil without manure-based fertilizer; CK0 is the soil with manure-based fertilizer at 0 mg/kg antibiotic concentration; OTC1 is the soil with manure-based fertilizer at 1 mg/kg OTC concentration; OTC10 shows the soil with manure-based fertilizer at 10 mg/kg OTC concentration; OTC100 is the soil with manure-based fertilizer at 100 mg/kg OTC concentration; SA1 is the soil with manure-based fertilizer at 1 mg/kg SA concentration; SA10 indicates the soil with manure-based fertilizer at 10 mg/kg SA concentration; SA100 is the soil with manure-based fertilizer at 100 mg/kg SA concentration. * or **represents significant differences ($p < 0.05$ or $p < 0.01$) between each antibiotic treatments (e.g., OTC1, OTC10) and CK0, and $p < 0.05$ or $p < 0.01$ on the line represents significant differences between treatment groups such as control, OTC, and SA treatments.
